# Adverse effects of maternal lead levels on birth outcomes in the ALSPAC study: a prospective birth cohort study

**DOI:** 10.1111/1471-0528.12756

**Published:** 2014-05-14

**Authors:** CM Taylor, J Golding, AM Emond

**Affiliations:** Centre for Child and Adolescent Health, School of Social and Community Medicine, University of BristolBristol, UK

**Keywords:** Birthweight, head circumference, lead, low birthweight, pregnancy, preterm

## Abstract

**Objective:**

To study the associations of prenatal blood lead levels (B-Pb) with pregnancy outcomes in a large cohort of mother–child pairs in the UK.

**Design:**

Prospective birth cohort study.

**Setting:**

Avon area of Bristol, UK.

**Population:**

Pregnant women enrolled in the Avon Longitudinal Study of Parents and Children (ALSPAC).

**Methods:**

Whole blood samples were collected and analysed by inductively coupled plasma dynamic reaction cell mass spectrometry (*n* = 4285). Data collected on the infants included anthropometric variables and gestational age at delivery. Linear regression models for continuous outcomes and logistic regression models for categorical outcomes were adjusted for covariates including maternal height, smoking, parity, sex of the baby and gestational age.

**Main outcome measures:**

Birthweight, head circumference and crown–heel length, preterm delivery and low birthweight.

**Results:**

The mean blood lead level (B-Pb) was 3.67 ± 1.47 *μ*g/dl. B-Pb ≥ 5 *μ*g/dl significantly increased the risk of preterm delivery (adjusted odds ratio [OR] 2.00 95% confidence interval [95% CI] 1.35–3.00) but not of having a low birthweight baby (adjusted OR 1.37, 95% CI 0.86–2.18) in multivariable binary logistic models. Increasing B-Pb was significantly associated with reductions in birth weight (*β* −13.23, 95% CI −23.75 to −2.70), head circumference (*β* −0.04, 95% CI −0.07 to −0.06) and crown–heel length (*β* −0.05, 95% CI −0.10 to −0.00) in multivariable linear regression models.

**Conclusions:**

There was evidence for adverse effects of maternal B-Pb on the incidence of preterm delivery, birthweight, head circumference and crown–heel length, but not on the incidence of low birthweight, in this group of women.

## Introduction

Lead is a neurotoxic metal that is widespread in the environment. The removal of lead from water pipes, paint and food cans, and a ban on lead additives to petrol in most countries has reduced exposure to lead in recent years, although industrial activities such as mining, smelting, lead shot manufacture and battery manufacture and recycling are still of concern. Food and drink, water, dust and soil remain as important sources of exposure,^1^ as does cigarette smoking.^2^,^3^

Lead readily crosses the placenta, with a fetal : maternal ratio of about 0.7–0.9,^4^–^6^ but studies on the association of blood lead levels in pregnancy and a variety of measures of birth outcomes, including birthweight (BW), head circumference (HC), gestational age at delivery and preterm delivery, have had inconsistent results at all levels of exposure.^7^–^11^ Poor birth outcomes are known to be associated with poor developmental trajectories in infancy, as well as having long-term implications for adult health.^12^–^14^ The effects of lead levels need to be characterised to enable delivery of appropriate public health policy and individual healthcare to lead-exposed women and newborn infants.

Previous studies have differed in their findings even in groups of women who have had high exposure to lead: for example, Factor-Litvak et al.^15^ found no effect on birth outcomes (BW, length of gestation, preterm delivery) despite a high blood lead level (B-Pb) of 19.1 *μ*g/dl in women living near a smelter in Kosovo, whereas McMichael et al.^16^ found an independent effect on preterm delivery, but not on low birthweight (LBW), in a similarly exposed group of women in South Australia (B-Pb 10.6 *μ*g/dl). In women who were not exposed to lead by their occupation or by nearby industrial processing, the outcomes have been similarly disparate.^8^,^17^–^19^ As has been discussed previously by Andrews et al. and Jelliffe-Pawlowski et al.,^9^,^10^ this could reflect between-study differences in sample timing, variation in the mean lead levels and therefore the extent of lead exposure, differences in statistical methods (for example, under- or over-control of covariates, and choice of categorical or continuous representation of predictors and outcomes in multiple regression), differences in sample sizes, and differences in the distributions of possible effect modifiers in different studies.

The aim of this study was to study the effects of B-Pb on birth outcomes—LBW, preterm delivery, BW, HC, crown–heel length (CHL)—in a group of mother–child pairs in the UK from the Avon Longitudinal Study of Parents and Children (ALSPAC).

## Methods

### The ALSPAC study

The sample was derived from the ALSPAC study, a population-based study investigating environmental and genetic influences on the health, behaviour and development of children. This database provided an opportunity to include a greater number of participants than has been reported on before and includes a wide range of social and demographic information to enable the most appropriate selection of covariates. All pregnant women in the former Avon Health Authority with an expected delivery date between 1 April 1991 and 31 December 1992 were eligible for the study; 14 541 pregnant women were enrolled initially, resulting in a cohort of 14 062 live births.^20^ The social and demographic characteristics of this cohort were similar to those found in UK national census surveys.^21^ Further details of ALSPAC are available at http://www.bris.ac.uk/alspac.

### Questionnaires

The mothers received four postal self-completion questionnaires during pregnancy. The questionnaires are available from the study website.^22^ Information on environmental and lifestyle factors included data on age, parity, social class, highest educational qualification, cigarette smoking, etc. Data on cigarette smoking were taken from a questionnaire completed at 18 weeks of gestation in response to a question on the number of cigarettes smoked in the previous 2 weeks (16–18 weeks of gestation).

### Pregnancy outcomes

Newborn HC and CHL were measured by trained study staff where the mother gave permission (*n* = 3280, 92.4%; *n* = 3224, 92.1%; respectively), or if these data were missing, the values were extracted from the medical records by trained study staff (*n* = 269, 7.6%; *n* = 275, 7.9%; respectively). BW was derived from obstetric data and from central birth notification data: where values disagreed by <100 g then the lowest value was accepted; if the values disagreed by >100 g then the value was coded as missing. Study staff were blinded to the maternal B-Pb. Pre-eclampsia was defined as systolic blood pressure >139 mmHg or diastolic blood pressure >89 mmHg measured on at least two occasions after 20 weeks of gestation with simultaneous proteinuria (dipstick test at least 1+; Albustix, Ames Co., Elkhart, IN, USA) (extracted from medical records by trained study staff). Length of gestation was based on last menstrual period date, ultrasound assessment or other clinical indicators. Where there was conflict between the maternal report and ultrasound assessment, an experienced obstetrician reviewed the clinical records and made a best estimate.

### Collection, storage and analysis of blood samples

Whole blood samples were collected in acid-washed vacutainers (Becton and Dickinson, Oxford, UK) by midwives as early as possible in pregnancy. Whole blood samples were stored in the original tube at 4°C at the collection site before being transferred to the central Bristol laboratory within 1–4 days. Samples were at ambient temperature during transfer (up to 3 hours). They were then stored at 4°C until analysis.

Details of the analysis have been reported.^23^ In brief, inductively coupled plasma mass spectrometry in standard mode (R. Jones, Centers for Disease Control, Bethesda, MD, USA; Method 3009.1) was used to measure blood levels of lead with appropriate quality controls.^17^

### Statistical analysis

Statistical analysis was done with spss version 19 (IBM Corp., Chicago, IL, USA). Values are reported as mean ± SD. Chi-square tests were used to analyse differences in categorical data, and two-sided *t* tests and were used to compare continuous values.

Univariable and multivariable linear regression models were used to examine the relationship of B-Pb on BW, HC and CHL. Logistic regression analysis was used to examine the effect of the binary variables preterm (<37 weeks of gestation) and LBW (<2500 g). Categories of <5 and ≥5 *μ*g/dl were chosen for the following reasons: (1) the US Association of Occupational and Environmental Clinics^24^ recommended in 2007 that B-Pb should be <5 *μ*g/dl during pregnancy, and this was endorsed by the recommendations of the US Centers for Disease Control in 2010^25^ and the American College of Obstetricians and Gynecologists in 2012;^26^ (2) 5 *μ*g/dl is the cut-off point recommended by the USA above which monitoring of children should be initiated.^27^ Factors included as confounders in the models were: maternal height, maternal prepregnancy weight, maternal educational attainment (none/Certificate of School Education, Vocational, O-levels, A-levels, degree), parity (0 versus ≥1), gestational age at delivery (weeks), number of cigarettes smoked per day and sex of the baby. These factors were broadly in accordance with those selected by Jelliffe-Pawlowski et al.^10^ as being consistently associated with BW, length of gestation, fetal growth retardation and other measures including LBW, preterm delivery and small-for-gestational-age at birth. However, unlike Jelliffe-Pawlowski et al.^10^ we did not include maternal ethnicity in any of the models because of the low numbers of ethnic minority participants included in the sample (non-white 2.4%). We also included smoking and maternal height and prepregnancy weight as these are also known to have associations with fetal growth.^28^,^29^ The linear regression analyses were repeated using log_10_ B-Pb to account for the skewed distribution of B-Pb. Regression diagnostics were used to check that the models fitted the observed data well and to identify any cases that had undue influence on the model.

## Results

The demographics of the subsample of the ALSPAC population have been reported previously.^2^ The subsample had slightly higher educational attainment (*P *= 0.004, chi-square test) and tended to be slightly older (*P *< 0.001, chi-square test) than the rest of the ALSPAC sample, but there were no other strongly statistically significant differences. The median gestational age at the time of blood sampling was 11 weeks (range 1–42 weeks, interquartile range 9–13 weeks). The analyses were completed on 4285 women for lead. One sample had a lead level below the limit of detection (0.29 *μ*g/dl): this sample was assigned a value of 0.7 times the lower limit of detection. The mean maternal B-Pb was 3.67 ± 1.47 *μ*g/dl (*n* = 4285; geometric mean 3.43, median 3.42, range 0.41–19.14 *μ*g/dl).^2^ The birth outcomes are summarised in Table 1.

**Table 1:** Birth outcomes and categorical associations of B-Pb (<5.00 or ≥5.00 *μ*g/dl) with birth outcomes

		B-Pb (*μ*g/dl)		*P* value
		<5.00	≥5.00	
Gestational age at delivery (weeks)	39.4 ± 2.1 (*n* = 4108)	39.4 ± 2.1 (*n* = 3516)	39.3 ± 2.3 (*n* = 592)	0.207*
Birthweight (g)	3411 ± 572 (*n* = 4052)	3424 ± 567 (*n* = 3469)	3334 ± 595 (*n* = 583)	0.001*
Head circumference (cm)	35.0 ± 1.5 (*n* = 3514)	34.8 ± 1.5 (*n* = 3010)	34.6 ± 1.8 (*n* = 504)	0.031*
Crown–heel length (cm)	50.7 ± 2.4 (*n* = 3467)	50.7 ± 2.3 (*n* = 2970)	50.4 ± 2.6 (*n* = 497)	0.011*
Low birthweight (<2500 g), *n* (%)	420/4269 (9.8%)	346/3654 (9.5%)	74/615 (12.0%)	0.048**
Preterm (<37 weeks), *n* (%)	238/3870 (5.8%)	186/3330 (5.3%)	52/540 (8.8%)	0.001**

Data are given as mean ± SD unless otherwise stated.

* Unpaired *t*-test.

** Chi-square test.

[Correction added on 12 December 2014 after first online publication: The values in the row heading, pre-term (<37 weeks), *n* (%) were miscalculated and have been corrected in Table 1 and results section.]

No cases were identified as outliers for the linear or the logistic regression models. BW, HC and CHL were each strongly significantly negatively linearly associated with B-Pb (*r* = −0.074, *P *< 0.001; *r* = −0.047, *P *= 0.006; *r* = −0.063, *P *< 0.001, respectively). B-Pb was not significantly greater in mothers delivering LBW (*n* = 420) versus not LBW (*n* = 3849) infants (3.79 ± 1.47 versus 3.65 ± 1.46 *μ*g/dl; *P *= 0.067, *t* test), or in those delivering preterm (*n* = 238) versus not preterm (*n* = 3870) (3.85 ± 1.66  *μ*g/dl vs 3.66 ± 1.45 *μ*g/dl; *P* = 0.053, *t* test). There was no significant difference in the B-Pb of those mothers having pre-eclampsia (*n* = 91) versus no pre-eclampsia (*n* = 3976) (3.63 ± 1.22 versus 3.67 ± 1.47; *P *= 0.806, *t* test).

Increasing B-Pb was strongly significantly associated with reductions in BW, HC and CHL (*P *< 0.001, *P *= 0.006 and *P *< 0.001, respectively) in univariable linear regression models (Table 2). These associations were maintained in multivariable models (*P *= 0.014, *P *= 0.021, *P *= 0.034, respectively). An increase of 1 *μ*g/dl in B-Pb predicted decreases in BW, HC and CHL of 13.2 g, 4 mm and 5 mm, respectively. The results were similar to those for the log values, so the results for non-log values are presented for ease of interpretation and comparison with other studies (see Table S1).

**Table 2:** Linear regression analysis: B-Pb (*μ*g/dl) as a predictor of birth outcomes

	*R*^2^ (%)	*β* (SE)	95% CI for *β*	*P* value
**Birthweight (g)**				
Univariate	0.7	−28.85 (6.10)	−40.82 to −16.88	<0.001
Multivariate*	38.7	−13.23 (5.37)	−23.75 to −2.70	0.014
**Head circumference (cm)**				
Univariate	0.2	−0.05 (0.02)	−0.08 to −0.01	0.006
Multivariate*	30.9	−0.04 (0.16)	−0.07 to −0.06	0.021
**Crown–heel (cm)**				
Univariate	0.4	−0.10 (0.03)	−0.16 to −0.05	<0.001
Multivariate*	32.1	−0.05 (0.03)	−0.10 to −0.00	0.034

All models overall *P *< 0.001.

* Adjusted for maternal height, maternal prepregnancy weight, maternal educational attainment, parity, no. of cigarettes per day, sex of baby, gestational age at delivery or death.

In univariable categorical analysis adverse birth outcomes (LBW and preterm delivery, lower BW, HC and CH) were strongly significantly associated with B-Pb ≥ 5.00 *μ*g/dl (*P *< 0.048, *P *= 0.001, *P = *< 0.001, *P = *0.021 and *P *= 0.011, respectively; *t*-test or chi-square test) (Table 1). Maternal B-Pb ≥ 5.00 *μ*g/dl increased the risk of preterm delivery (adjusted odds ratio 2.00, 95% confidence interval 1.35–3.00) but not of having an LBW infant (adjusted odds ratio 1.37, 95% confidence interval 0.86–2.18) in multivariable logistic multiple regression analysis (Table 3).

**Table 3:** Logistic regression analysis: B-Pb <5 (reference) or ≥5 *μ*g/dl as a predictor of preterm delivery and of low birthweight

	OR	95% CI	*P* value
**Preterm (yes)**			
Univariate	1.72	1.25–2.38	0.001
Multivariate*	2.00	1.35–3.00	0.001
**LBW (yes)**			
Univariate	1.31	1.00–1.71	0.049
Multivariate**	1.37	0.86–2.18	0.187

Preterm, <37 weeks of gestation; low birthweight (LBW), <2500 g.

* Adjusted for maternal height, maternal prepregnancy weight, maternal educational attainment, parity, no. of cigarettes per day, sex of baby.

** Adjusted for maternal height, maternal prepregnancy weight, maternal educational attainment, parity, no. of cigarettes per day, sex of baby and gestational age at delivery or death.

[Correction added on 12 December 2014 after first online publication: The pre-term univariate and multivariate values were miscalculated and have been corrected in Table 3 and results section.]

## Discussion

### Main findings

This study provides evidence for adverse effects of maternal B-Pb on pregnancy outcomes. Based on the premise that the fetal B-Pb is about 80% of that of the mother, US recommendations are that maternal B-Pb should be kept <5 *μ*g/dl;^24^–^26^ using this value as a cut-off, we have shown an increased risk of preterm delivery at maternal levels ≥5.00 *μ*g/dl but there was no effect of maternal B-Pb on LBW. We have also shown reductions in BW, HC and CHL with increasing maternal B-Pb.

### Strengths and limitations

The strength of our study lies in the large number of pregnant women that we were able to include, which would help to highlight subtle effects. The limitations of our study were the bias in the sample (women who had B-Pb measurements were older and had higher educational levels than the rest of the ALSPAC cohort). This would not be expected to affect the independent results of regression and logistic models, however. It is likely that maternal B-Pb in the UK will have fallen further in recent years, although it is possible that exposure levels may remain relatively high in the Bristol area of the UK due to a local history of lead mining and working.^2^,^30^ Finally, it should be noted that this study shows associations only and it is not possible to take all confounders into account in the models.

### Interpretation

The mechanisms by which lead could have an adverse effect on pregnancy outcomes include an impairment of fetal bone growth caused by competition with calcium for deposition into bone,^31^ reductions in fetal thyroid hormones,^32^ accumulation of lead in the placenta causing abnormal placental function and reduced nutrient transfer,^33^ and oxidative stress.^34^ Preterm delivery may be prompted by premature rupture of the membranes through lead-induced reactive oxygen species causing collagen damage and hence structural weakness.^19^ There is considerable controversy in the literature, however, concerning the effects of maternal B-Pb on pregnancy outcomes, even in groups of mothers with relatively high exposures to lead (see Table S2). It has been proposed that these differing findings result from methodological differences.^9^,^10^ These include differences in the biological material used to measure the lead level, the timing of the samples, the range of lead levels in the samples, the timing of any specific lead exposure, and substantial differences in the methods used for statistical analysis, including choice of confounders, under- and over-control of confounders, and the use of categorical or continuous data. In addition, a range of outcomes have been chosen, and sample sizes have sometimes been very small (see Table S2). These variations are not always easy to identify in the literature, which makes comparisons of results between different studies difficult.^9^ To address as many of these concerns as possible, we were able to include a large number of pregnant women in our sample. The median gestational age at which the blood sample was taken was 11 weeks: as B-Pb are thought to follow a U-shaped curve during pregnancy,^35^ this makes comparison of levels in studies in which blood sample were taken in the first with the third trimester valid. Following the conclusions of Andrews et al.,^9^ we excluded alcohol intake as a confounder but included cigarette smoking, and present our results as univariable and multivariable. We used a set of confounders that were very similar to those of Jelliffe-Pawlowski et al.,^10^ except that we excluded adjustment for ethnicity because our sample included very few women from ethnic minorities.

It might be expected therefore that populations with high exposure would have the greatest risk of adverse pregnancy outcomes: high maternal B-Pb or occupational exposure have been associated with preterm delivery,^16^ miscarriage,^36^ small-for-gestational-age babies,^37^ and effects on BW and the incidence of neural tube defects.^38^ However, other studies have not observed any effects in mothers with high exposure levels.^15^,^39^–^41^ The findings of studies where B-Pb were relatively low have been equally disparate. For example, linear associations with premature delivery,^18^ and an association with the length of gestation but not with BW or HC^17^ have been found at mean B-Pb < 5 *μ*g/dl. Two recent studies, however, have converged on finding effects on BW but not on other pregnancy outcomes despite a large between-study difference in maternal B-Pb.^7^,^8^

The decrease in BW of 13.2 g per increase of 1 *μ*g/dl in B-Pb found in the present study is greater than has been reported previously,^7^,^32^,^42^ even though the B-Pb reported in other studies tended to be higher than that in the present study (mean B-Pb in present study 3.67 ± 1.47 *μ*g/dl versus, for example, mean maternal B-Pb 8.9 *μ*g/dl^42^). However, it has been suggested that the adverse effects are relatively greater at lower B-Pb: in a large study (42 288 mother–infant pairs from a US registry) in which only B-Pb < 10 *μ*g/dl were included, the decrease ranged from 4 g for a change from 9 to 10 *μ*g/dl to 27 g for a change from 0 to 1 *μ*g/dl.^7^ These values were stated as being consistent with other studies in which decreases ranging from 0.3 g^32^ to 6.2 g^42^ per 1 *μ*g/dl increase in B-Pb were reported.

The decreases of 4 mm in HC and 5 mm in CHL per increase of 1 *μ*g/dl in B-Pb found in the present study were small but nonetheless statistically significant. Associations of B-Pb with CHL and HC have not generally been found in previous studies^8^,^11^,^17^,^41^,^43^ even when the mean B-Pb was relatively high,^41^ although this is not a consistent finding^44^,^45^ (see Table S2).

The effects of B-Pb on preterm delivery (or length of gestation) and LBW (or small-for-gestational-age) are similarly divided in the literature, with some authors reporting no effect on one or both of the variables,^7^,^8^,^46^,^47^ and others reporting linear associations,^16^,^18^ or a doubling^37^ or even trebling^10^ of risk for B-Pb > 10 *μ*g/dl (see Table S2). In line with these findings, but using a lower cut-off level of 5 *μ*g/dl, we found that the risk of preterm delivery in women with levels ≥ 5 *μ*g/dl was twice that in women with levels < 5 *μ*g/dl.

## Conclusion

There was an adverse effect of B-Pb on pregnancy outcomes in this group of women, with reductions in BW, HC and CHL with increasing B-Pb, and an almost twofold increased risk of preterm delivery for maternal B-Pb > 5 *μ*g/dl. LBW was not associated with maternal B-Pb. These adverse effects may have important long-term effects on the physical and neurological development of the child and into adulthood: further work will be undertaken in the ALSPAC cohort to study the effects of maternal B-Pb on the growth and development of the child up to 18 years.

## Supporting Information

Table S1 Linear multiple regression: log10 B-Pb (*μ*g/dl) as predictor of birth outcomes. **Table S2.** Summary of studies on associations between maternal B-Pb and pregnancy outcome.
