# Supplementary material for: Adverse effects of maternal lead levels on birth outcomes in the ALSPAC study: a prospective birth cohort study
Source: BJOG. 2014 May 14;122(3):322–8. doi: 10.1111/1471-0528.12756 (PMC4322474; doi:10.1111/1471-0528.12756)
Supplement: Table S1 — Linear multiple regression: log10 B-Pb (μg/dl) as predictor of birth outcomes. Table S2. Summary of studies on associations between maternal B-Pb and pregnancy outcome. [file bjo0122-0322-sd1.pdf]

Supplementary Table S1 Linear multiple regression: $\log_{10}$ B-Pb (µg/dl) as predictor of birth outcomes

	R^2 (%)	B (SE)	95% CI for B	p value
Birth weight (g)				
Univariate	0.6	–274.18 (31.21)	–383.92 to –164.44	<0.001
Multivariate[a]	38.7	–120.19 (48.37)	–215.02 to –25.36	0.013
Head circumference (cm)				
Univariate	0.2	–0.48 (0.16)	–0.80 to –0.16	0.003
Multivariate[a]	30.9	–0.35 (0.15)	–0.63 to –0.06	0.019
Crown–heel (cm)				
Univariate	0.4	–0.95 (0.25)	–1.47 to –0.45	<0.001
Multivariate[a]	32.1	–0.50 (0.23)	–0.95 to –0.05	0.031

[a]Adjusted for maternal height, maternal prepregnancy weight, maternal educational attainment, parity, no of cigarettes per day, sex of baby, gestational age at delivery or death.

All models overall p<0.001.

Supplementary Table S2 Summary of studies on associations between maternal B-Pb and pregnancy outcomes

Authors	Location of study	n	Mean maternal B-Pb (μg/dl)	Pregnancy outcome(s) studied	Results	Comments
**Low exposure[a]**						
Xie et al., 2013[1]	China	252	1.00–11.91, median 3.2	Birth length<br>BW<br>HC	Association of B-Pb with BW<br>No association with HC or birth length	
Vigeh et al., 2011[2]	Tehran, Iraq	348	3.8 (1.0–20.5), geometric mean 3.5	Preterm delivery	B-Pb higher in women who delivered preterm babies<br>Association of B-Pb with preterm delivery	
Gundacker et al., 2010[3]	Vienna, Austria	53	2.5	BW<br>Birth length<br>Head circumference	Association of B-Pb with birth weight<br>No association with length of gestation or head circumference	
Vigeh et al., 2010[4]	Tehran, Iraq	332	PROM: 4.61±2.37 (n=36)<br>Not PROM: 3.69±1.85 (n=296)	PROM	B-Pb higher in women who delivered babies with PROM<br>Association of B-Pb with PROM	
**High exposure[b]**						
Rahman et al., 2012[5]	Kuwait	194	5.77	BW<br>Crown–heel length<br>Head circumference<br>APGAR score<br>Gestational age<br>Placental weight	No associations with B-Pb<br>Cord B-Pb associated with APGAR score in boys only	
Zhu et al., 2010[6]	New York	45,000	Selected those with B-Pb <10	Preterm birth<br>SGA<br>LBW	Small risk of decreased BW with increasing B-Pb<br>No association with preterm birth or SGA	State database
Mirghani, 2010[7]	Saudi Arabia (Aseer region)	128	<20 (n=86)<br>>20 (n=41)	BW<br>Gestational age<br>PROM	No associations	
Chen et al., 2006[8]	Taiwan	1611	10.1±10.4	SGA	Association of B-Pb >20 with SGA	Occupational exposure database
Rahman and Hakeem, 2003[9]	Karachi, Pakistan	73	9.91±4.44 (2.28–36.35)	Gestational age<br>BW<br>Recumbent length<br>Head circumference	No associations<br>Mothers of boys had higher B-Pb than mothers of girls	

Bjerregaard and Hansen, 2000[10]	Disko Bay, Greenland	180	3.57±4.15	BW<br>Length	No associations	High consumption of marine meat and blubber
Factor-Litvak et al., 1999[11]	Kosovo, Yugoslavia	401	Exposed to smelter: 19.1±7.9<br>Not exposed: 5.6±1.9	Length of gestation<br>BW<br>Preterm birth	No associations	
Borja-Aburto et al., 1999[12]	Mexico City	668	Cases: 12.03<br>Controls: 10.09	Spontaneous abortion	Increased risk of spontaneous abortion	
Gonzales-Cossio et al., 1997[13]	Mexico	272	8.9±4.1 (median 8.1)<br>Tibia and patella lead levels	BW	Association of tibia lead with BW<br>No associations for maternal B-Pb or patella lead	
West et al., 1994[14]	USA	349	6.36±0.19 (2.7–12.0)	Gestational age<br>LBW<br>BW	Association of gestation age with B-Pb<br>No association for BW<br>No association for LBW unless excluded women with genital infections.	African-American women
McMichael et al., 1986[15]	Port Pirie, South Australia	749	Smelter community: 10.6<br>Non-smelter community: 7.6	Preterm delivery<br>Miscarriage<br>LBW at term<br>IUGR<br>PROM<br>Congenital anomalies	Association of preterm delivery with B-Pb<br>No other associations	
**Miscellaneous**[c]						
Atabek et al., 2007[16]	Turkey	54	14.4 ±0.89 (5.1–35.5) (cord blood)	BW<br>Other anthropometric variable	Association of B-Pb with BW<br>No association with any other variables	
Berkowitz et al., 2006[17]	Idaho, USA	169,878	–	Preterm birth<br>SGA<br>LBW<br>BW	Associations of living in accident area at time of accident with BW, SGA and BW<br>No association with preterm birth	Mothers living in area of smelter which had accident in 1973 resulting in high air lead levels for 6 months
Zentner et al., 2006[18] [d]	Brazil	55	– (cord blood)	BW<br>Length	Associations of B-Pb with BW and length	
Wang et al., 2004[19]	China	89	Cord blood levels<br>Rural: 7.4 [a] (3.52–24.6)<br>Industrial: 6.59[a] (2.0–17.2)<br>Urban: 5.43[a] (2.79–18.7)	BW<br>Birth length	No associations	

Falcon et al., 2003[20]	Spain	89	113.4±58.0 mg/g dry weight (placenta lead)	BW<br>Head circumference<br>Abdominal circumference<br>Length	No associations	
Yao and Huang, 2003[21] [d]	Beijing, China	1151	4.34	BW<br>Length<br>HC<br>Chest circumference<br>Abdomen circumference<br>Neurobehavioural development at 42 days<br>Pregnancy complications	No associations	
Sowers et al., 2002[22] [d]	USA	705	1.2±0.03	APGAR score<br>BW<br>Gestational age<br>SGA	No associations	
Durska, 2001[23] [d]	Szczecin, Poland	83	1.33	BW<br>Length<br>HC<br>Chest circumference	No associations	
Osman et al., 2000[24]	Sweden	106	26 (0–630) nmol/kg (placenta lead)<br>54 nmol/l (cord blood; equivalent to 1.12 µg/dl)	BW<br>Head circumference<br>Length	Association of cord B-Pb with birth length, head circumference and BW	
Richter et al., 1999[25] [d]	Czech Republic	Control group: 50<br>IUGR group: 43	–	IUGR	Lower B-Pb in IUGR group	
Irgens et al., 1998[26]	Norway	Maternal (n=1886) or paternal (n=35,802) occupational lead exposure vs no exposure	–	LBW<br>Gestational age<br>Preterm birth<br>Neural tube defects and other birth defects<br>Perinatal mortality<br>Down’s syndrome	Increased risk of LBW and neural tube defects in occupationally exposed mothers<br>Decreased risk of LBW and preterm birth for occupationally exposed fathers	
Philion et al., 1997[27]	Canada	9329	Living in smelter city vs not	SGA	No association of exposure on SGA	Birth records 1961–

						1990
Fagher et al., 1993[28 d]	Sweden/Poland	Preterm delivery: 17<br>Term delivery: 13	Polish women: 3.79<br>Swedish women: 1.12	Preterm delivery	No effect of lead causing increase in myometrial activity	
Bellinger et al., 1991[29]	Australia	4354	7.0±3.3 (cord blood)	Length of gestation<br>BW<br>SGA	No adverse effects on fetal growth at levels <15, but may be modest effects >15	

Adapted with permission from Taylor et al. (2014).[30]

[a]Mean or median maternal B-Pb <5 μg/dl; [b]mean or median maternal B-Pb ≥5 μg/dl.

[c]Categorised as miscellaneous because: (1) measurement of lead in tissue other than maternal blood; (2) measure of lead level not reported; (3) abstract only retrieved.

[d]Abstract only retrieved.

B-Pb, blood lead level; BW, birth weight; IUGR, intrauterine growth retardation; LBW, low birth weight; PROM, premature rupture of the membranes; SGA, small for gestational age.

References

1. Xie X, Ding G, Cui C, Chen L, Gao Y, Zhou Y, et al. The effects of low-level prenatal lead exposure on birth outcomes. *Environ Pollut*. 2013;175:30-34
2. Vigeh M, Yokoyama K, Seyedaghamiri Z, Shinohara A, Matsukawa T, Chiba M, et al. Blood lead at currently acceptable levels may cause preterm labour. *Occup Environ Med*. 2011;68:231-234
3. Gundacker C, Frohlich S, Graf-Rohrmeister K, Eibenberger B, Jessenig V, Gicic D, et al. Perinatal lead and mercury exposure in Austria. *Sci Total Environ*. 2010;408:5744-5749
4. Vigeh M, Yokoyama K, Shinohara A, Afshinrokh M, Yunesian M. Early pregnancy blood lead levels and the risk of premature rupture of the membranes. *Reprod Toxicol*. 2010;30:477-480
5. Rahman A, Al-Rashidi HA, Khan AR. Association of maternal blood lead level during pregnancy with child blood lead level and pregnancy outcome in Kuwait. *Ecol Food Nutr*. 2012;51:40-57
6. Zhu M, Fitzgerald EF, Gelberg KH, Lin S, Druschel CM. Maternal low-level lead exposure and fetal growth. *Environ Health Persp*. 2010;118:1471-1475

7. Mirghani Z. Effect of low lead exposure on gestational age, birth weight and premature rupture of the membrane. *J Pakistan Med Assoc*. 2010;60:1027-1030
8. Chen PC, Pan IJ, Wang JD. Parental exposure to lead and small for gestational age births. *Am J Industr Med*. 2006;49:417-422
9. Rahman A, Hakeem A. Blood lead levels during pregnancy and pregnancy outcome in Karachi women. *J Pakistan Med Assoc*. 2003;53:529-533
10. Bjerregaard P, Hansen JC. Organochlorines and heavy metals in pregnant women from the Disko Bay area in Greenland. *Sci Total Environ*. 2000;245:195-202
11. Factor-Litvak P, Graziano JH, Kline JK, Popovac D, Mehmeti A, Ahmedi G, et al. A prospective study of birthweight and length of gestation in a population surrounding a lead smelter in Kosovo, Yugoslavia. *Int J Epidemiol*. 1991;20:722-728
12. Borja-Aburto VH, Hertz-Picciotto I, Rojas Lopez M, Farias P, Rios C, Blanco J. Blood lead levels measured prospectively and risk of spontaneous abortion. *Am J Epidemiol*. 1999;150:590-597
13. Gonzalez-Cossio T, Peterson KE, Sanin LH, Fishbein E, Palazuelos E, Aro A, et al. Decrease in birth weight in relation to maternal bone-lead burden. *Pediatrics*. 1997;100:856-862
14. West WL, Knight EM, Edwards CH, Manning M, Spurlock B, James H, et al. Maternal low level lead and pregnancy outcomes. *J Nutr*. 1994;124:981S-986S
15. McMichael AJ, Vimpani GV, Robertson EF, Baghurst PA, Clark PD. The port pirie cohort study: Maternal blood lead and pregnancy outcome. *J Epidemiol Commun Health* 1986;40:18-25
16. Atabek ME, Kurtoglu S, Pirgon O, Uzum K, Saraymen R. Relation of in utero lead exposure with insulin-like growth factor-i levels and neonatal anthropometric parameters. *Int J Hyg Environ Health*. 2007;210:91-95
17. Berkowitz Z, Price-Green P, Bove FJ, Kaye WE. Lead exposure and birth outcomes in five communities in Shoshone county, Idaho. *Int J Hyg Environ Health* 2006;209:123-132
18. Zentner LE, Rondo PH, Mastroeni SS. Lead contamination and anthropometry of the newborn baby. *J Trop Pediatr*. 2006;52:369-371.
19. Wang C, Huang L, Zhou X, Xu G, Shi Q. Blood lead levels of both mothers and their newborn infants in the middle part of China. *Int J Hyg Environ Health*. 2004;207:431-436
20. Falcon M, Vinas P, Luna A. Placental lead and outcome of pregnancy. *Toxicology*. 2003;185:59-66
21. Yao HY, Huang XH. The blood lead level and pregnant outcome in pregnant women with non-occupational lead exposure. *Chinese J Obstetr Gynaecol*. 2003;38:340-342
22. Sowers M, Jannausch M, Scholl T, Li W, Kemp FW, Bogden JD. Blood lead concentrations and pregnancy outcomes. *Arch Environ Health*. 2002;57:489-495
23. Durska G. [Levels of lead and cadmium in pregnant women and newborns and evaluation of their impact on child development]. *Annales Academiae Medicae Stetinensis*. 2001;47:49-60
24. Osman K, Akesson A, Berglund M, Bremme K, Schutz A, Ask K, et al. Toxic and essential elements in placentas of Swedish women. *Clin Biochem*. 2000;33:131-138
25. Richter J, Hájek Z, Pfeifer I, Šubrt P. Relation between concentration of lead, zinc and lysozyme in placentas of women with intrauterine foetal growth retardation. *Cent Eur Public Health*. 1999;7:40-42

26. Irgens A, Kruger K, Skorve AH, Irgens LM. Reproductive outcome in offspring of parents occupationally exposed to lead in Norway. *Am J Industr Med*. 1998;34:431-437
27. Philion JJ, Schmitt N, Rowe J, Gelpke PM. Effect of lead on fetal growth in a Canadian smelter city, 1961-1990. *Arch Environ Health*. 1997;52:472-475
28. Fagher U, Laudanski T, Schutz A, Sipowicz M, Akerlund M. The relationship between cadmium and lead burdens and preterm labor. *Int J Gynaecol Obstetr*. 1993;40:109-114
29. Bellinger D, Leviton A, Rabinowitz M, Allred E, Needleman H, Schoenbaum S. Weight gain and maturity in fetuses exposed to low levels of lead. *Environ Res*. 1991;54:151-158
30. Taylor CM, Golding J, Emond AE. Lead, cadmium and mercury levels in pregnancy: the need for international consensus on levels of concern. *J Dev Origins Health Dis.* 2014;5:16-30.
